# High Grade Dermatologic Adverse Events Associated With Immune Checkpoint Blockade for Cancer

**DOI:** 10.3389/fmed.2022.898790

**Published:** 2022-06-13

**Authors:** Alyce M. Kuo, Alina Markova

**Affiliations:** ^1^Dermatology Service, Department of Medicine, Memorial Sloan Kettering Cancer Center, New York, NY, United States; ^2^Department of Dermatology, Weill Cornell Medical College, New York, NY, United States

**Keywords:** toxic epidermal necrolysis (TEN), Stevens-Johnson syndrome (SJS), immune checkpoint blockade (ICB), acute generalized exanthematous pustulosis (AGEP), drug reaction with eosinophilia and systemic symptoms (DRESS), bullous pemphigoid (BP), rash, dermatologic adverse events (DAEs)

## Abstract

Immune checkpoint blockade (ICB) improves survival in many types of cancers including melanoma, non-small cell lung, renal cell, breast, and cervical cancers. However, many of these therapies are also associated with high grade dermatologic adverse events (DAEs), including Stevens-Johnson syndrome and toxic epidermal necrolysis (SJS/TEN), SJS/TEN-like reactions, high grade maculopapular and psoriasiform rashes, autoimmune bullous eruptions, drug reaction with eosinophilia and systemic symptoms (DRESS), and acute generalized exanthematous pustulosis (AGEP), which may limit their tolerability and use. It is important to properly identify and treat DAEs to ICB because these DAEs may be associated with positive anti-tumor response and patients may have limited options for alternative anti-cancer therapeutics. In this review, we describe high grade DAEs to increasingly used ICB agents, which target CTLA-4 and PD-1 or its ligand, PD-L1 and enable the immune system to target cancer cells. We further differentiate life-threatening adverse reactions from mimickers and report cases of serious DAEs which have been recorded in association with ICB through the FDA Adverse Events Reporting System (FAERS), which is an archive of adverse events associated with various drugs and therapeutic biologic products reported voluntarily by consumers and healthcare professionals as well as mandatorily by manufacturers. Lastly, we summarize management recommendations for these adverse events and discuss knowledge and evidence gaps in this area.

## Introduction

Immune checkpoint blockade (ICB) improves survival in many cancers including melanoma, non-small cell lung, renal cell, breast, and cervical cancers (1–5). However, it is also associated with dermatologic adverse events (DAEs), which may limit its tolerability and use. Although most DAEs are mild or moderate, others may be systemic and even life-threatening. High grade [Common Terminology Criteria for Adverse Events (CTCAE) grade ≥3, see Table 1] (6) DAEs cover a spectrum of entities, including Stevens-Johnson syndrome and toxic epidermal necrolysis (SJS/TEN), SJS/TEN-like reactions, bullous eruptions, drug reaction with eosinophilia and systemic symptoms (DRESS), and acute generalized exanthematous pustulosis (AGEP) (7). It is important to properly identify and treat DAEs to ICB because patients often have limited options for alternative anti-cancer therapeutics. In this review, we describe high grade DAEs to ICB, differentiating life-threatening DAEs from mimickers. We also report cases of serious DAEs which have been recorded in association with ICB through the FDA Adverse Events Reporting System (FAERS), which is an archive of adverse events associated with various drugs and therapeutic biologic products reported voluntarily by consumers and healthcare professionals as well as mandatorily by manufacturers.

**TABLE 1:** DAE grading (Adapted from the CTCAE Version 5.0) (6).

DAE	Grade	Description
SJS/TEN	3	Skin sloughing <10% body surface area (BSA) + associated signs (mucous membrane detachment, etc.)
	4	Skin sloughing 10–30% BSA (SJS) or ≥30% BSA (TEN) + associated signs
Rash maculopapular	1	Macules/papules covering <10% BSA ± symptoms (pruritus, burning, etc.)
	2	Macules/papules covering 10–30% BSA ± symptoms (pruritus, burning, etc.), limiting instrumental activities of daily living (ADL), or ≥30% BSA ± mild symptoms
	3	Macules/papules covering >30% BSA + moderate/severe symptoms, limiting self-care ADL
Bullous dermatitis	1	Asymptomatic, blisters covering <10% BSA
	2	Blisters covering 10–30% BSA, painful blisters, or limiting instrumental ADL
	3	Blisters covering >30% BSA, limiting self-care ADL
	4	Blisters covering >30% BSA + fluid/electrolyte abnormalities, ICU/burn unit indicated
	5	Death
Other skin disorders (Other DAEs)	1	Asymptomatic or mild symptoms
	2	Moderate; limiting ADL
	3	Severe or medically significant but not life threatening
	4	Life-threatening consequences
	5	Death

## Immune Checkpoint Blockade

The anti-CTLA-4 monoclonal antibody ipilimumab, anti-PD-1 monoclonal antibodies cemiplimab, nivolumab, and pembrolizumab, and anti-PD-L1 monoclonal antibodies atezolizumab, avelumab, and durvalumab overcome immune checkpoints, allowing the immune system to target cancer cells. These agents are associated with many immune-related DAEs (irDAEs), which tend to develop earlier than non-cutaneous immune-related adverse events (irAEs) (8, 9). Although the most common irDAEs to ICB, such as maculopapular rash, pruritus, and lichenoid dermatoses, may be controlled with topical corticosteroids and oral anti-pruritics, high grade irDAEs may require prolonged systemic therapy and/or discontinuation of the culprit immunotherapy (10). Importantly, the development of irDAEs has been associated with better overall survival in patients treated with ICB (11).

## True Stevens-Johnson Syndrome and Toxic Epidermal Necrolysis

True SJS/TEN has been described in association with ICB, with classic rapid onset and progression and high mortality rates ranging from 10% for SJS to 50% for TEN (10, 12). As of March 2022, 255 cases of SJS/TEN had been reported through FAERS with pembrolizumab, 102 with ipilimumab, 224 with nivolumab, 55 with atezolizumab, 3 with avelumab, 21 with durvalumab, and 4 with cemiplimab. Diagnosis of true SJS/TEN is based on mucocutaneous involvement with supportive histopathological findings. Irregularly shaped dark, dusky macules may spread from the trunk and proximal extremities to the rest of the body. Patients may first present with a prodrome of malaise, followed by mucocutaneous pain as mucosal membranes and skin undergo necrolysis, upper respiratory symptoms, and fever, later developing systemic involvement of the liver, lungs, or gastrointestinal tract (13). Biopsy typically reveals full thickness epidermal necrosis with vacuolar interface changes, cleavage along the dermal epidermal junction, and subepidermal lymphocytes (14).

When SJS/TEN is suspected, urgent dermatologic evaluation is necessary and inpatient admission should be considered and ICB as well as other potential culprit medications should be held (15). Those with widespread mucocutaneous desquamation or life-threatening complications should be admitted to the intensive care or burn unit (16). Skin biopsies should be assessed for full-thickness epidermal necrosis, which is seen in true SJS/TEN and SJS/TEN-like reactions. Management of true SJS/TEN in patients on ICB must include supportive care and ophthalmologic, gynecologic and/or urologic consultations depending on extent and location of mucosal involvement. ICB must be discontinued once true ICB-associated SJS/TEN diagnosis is confirmed. National Comprehensive Cancer Network (NCCN) Guidelines for Management of Immunotherapy-Related Toxicities (version 1.2022) (15) provide recommendations for SJS/TEN management (without differentiating the treatment for both true SJS/TEN and SJS/TEN-like rashes) with prednisone or methylprednisolone 1–2 mg/kg/day and intravenous immune globulin (IVIg) 1 g/kg/day and/or other immunosuppressive therapies, including etanercept and cyclosporine can be considered for true SJS/TEN (15).

## Stevens-Johnson Syndrome and Toxic Epidermal Necrolysis-Like Reactions

**FIGURE 1:**
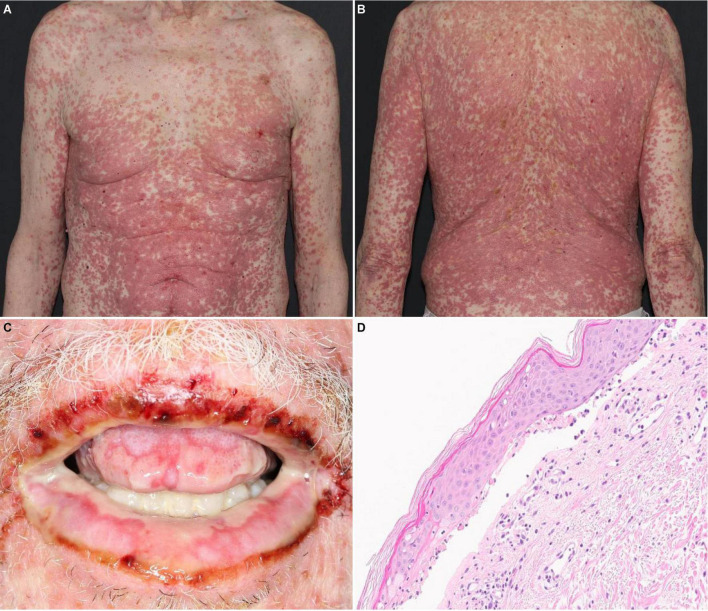
SJS/TEN-like reaction with erythematous macules and papules with dusky, purpuric centers covering about 80% BSA on the **(A)** trunk and **(B)** back. **(C)** Desquamation and hemorrhagic crusts on the oral mucosa. **(D)** Histology revealing lichenoid and subepidermal vesicular dermatitis with epidermal necrosis.

Incidence of SJS/TEN-like reactions is not known. Because cases of SJS/TEN in the FAERS database are voluntarily reported and unverified, they likely include SJS/TEN-like reactions, which mimic SJS/TEN but vary in severity and clinical course. While ipilimumab has not independently been associated with SJS/TEN-like reactions, emerging evidence suggests anti-PD-1/PD-L1 therapies are associated more frequently with SJS/TEN-like reactions (Figures 1A–C) than true SJS/TEN (17, 18). Unlike true SJS/TEN, which presents acutely, some SJS/TEN-like reactions to anti-PD-1/PD-L1 blockade progress from mild DAEs over a few to several weeks. Initially, patients may present with a morbilliform eruption, which then turn into targetoid patches and epidermal detachment with associated mucositis. Alternatively, other SJS/TEN-like reactions occur *de novo* late in the course of treatment with anti-PD-1/PD-L1 therapy. In one series of 18 patients, 2 developed SJS/TEN-like reactions *de novo* without preceding rash more than 6 weeks after initiating treatment with anti-PD-1/PD-L1 blockade (19). These reactions develop weeks to months after initiating treatment (median: 52 days, range: 3–420 days) (14, 20). SJS/TEN-like reactions due to pembrolizumab typically occur later with a median onset of 11 weeks and average of 12.8 weeks after initiation (19). SJS/TEN-like reactions present with a more benign clinical course and favorable treatment response when compared to true SJS/TEN (18). However, concurrent use of multiple ICB agents such as ipilimumab with nivolumab can lead to earlier and more severe DAEs, as seen in one analysis of pooled safety data from 1,551 patients with advanced melanoma (21). SJS/TEN-like reactions may occur concurrently with extra-cutaneous irAEs. In a pooled analysis of three trials of 448 patients with advanced melanoma who received ipilimumab/nivolumab, the most frequently reported irAEs involved skin (64.3%) and GI (46.7%). Thirty percent of patients developed grade 2–4 irAEs in more than one organ system (22).

Antibiotic use may precipitate SJS/TEN and SJS/TEN-like reactions to ICB. A large retrospective study of 767 patients treated with ICB at a single institution and analysis of 38,705 safety reports of patients receiving anti-PD-1/PD-L1 from FAERS found that irAE potential risks including SJS/SJS-like development was higher in patients who used antibiotics during ICB therapy compared to those who did not (23). ICB may also increase a patient’s risk of developing SJS/TEN and SJS/TEN-like reactions to other agents. One series of seven patients who developed SJS-like reactions after anti-PD-1/PD-L1 with or without anti-CTLA-4 blockade found that all patients had received newly initiated drugs such as trimethoprim-sulfamethoxazole and allopurinol before DAE onset. A 2-hit hypothesis may play a part in the explanation for this association: ICB may first modulate the immune system to heighten drug sensitivity and then addition of a second drug/agent can then trigger an SJS-like reaction (18). Therefore, it is important to carefully identify the culprit agent and to differentiate SJS/TEN-like reactions from true SJS/TEN to potentially allow patients to continue therapy with ICB. Interestingly, even after discontinuation of ICB, patients are still at risk for SJS/TEN-like reactions (24, 25). This may be due to the long half-life of ICB and persistent immune activation in the setting of prolonged tumor responses, which has been observed with both anti-PD-1 and anti-CTLA-4 therapy (26).

Although SJS/TEN-like reactions resemble true SJS/TEN on histopathology (Figure 1D), with characteristic findings such as full-thickness epidermal necrolysis, subepidermal clefting, and interface dermatitis, severe clinical symptoms such as fever, ocular involvement, and maximal detachment are much rarer and seen in as few as 8% of patients (17).

In the setting of SJS/TEN-like reactions, ICB should initially be held along with other potential culprit medications. Wound care, topical emollients and high-strength topical steroids can be started (27). NCCN Guidelines for Management of Immunotherapy-Related Toxicities (version 1.2022) for SJS/TEN management (without differentiating the treatment for both true SJS/TEN and SJS/TEN-like rashes) with prednisone or methylprednisolone 1–2 mg/kg/day and IVIg 1 g/kg/day and/or other immunosuppressive therapies, including etanercept and cyclosporine can be considered for true SJS/TEN (15). While etanercept, cyclosporine, and/or IVIg are preferred for true SJS/TEN, topical and systemic steroids are typically used as first-line for SJS/TEN-like eruptions; use of cyclosporine, IVIg, and/or targeted therapies including etanercept, infliximab, tocilizumab, dupilumab may also be considered (27–29). For SJS/TEN-like eruptions, rechallenge of ICB may be considered once all skin and extracutaneous involvement resolves to grade ≤1, following a multidisciplinary discussion taking into consideration DAE severity, any required concurrent immunosuppressant for DAE management, prior cancer response to ICB, and alternative anti-cancer therapies (18, 30).

## High-Grade Maculopapular Rashes

Pruritic, maculopapular rashes are among the most frequent DAEs associated with ICB (10). High grade (grade 3) maculopapular rashes covering >30% of total body surface area, which develop a median of 3.6 weeks after initiation of anti-CTLA-4 blockade, have been observed in up to 4% of patients (31). There are 1,190 reported cases of serious maculopapular rashes with pembrolizumab, 1,340 with ipilimumab, 1,934 with nivolumab, 385 with atezolizumab, 32 with avelumab, 122 with durvalumab, and 36 with cemiplimab recorded in FAERS. These maculopapular rashes typically present with numerous coalescing macules and papules and most often affects the trunk and extremities (10). Biopsy reveals interface and perivascular/periadnexal lymphocytic dermatitis with or without eosinophils (32). For high grade maculopapular rashes, NCCN guidelines recommend initial management with holding ICB and applying high potency topical steroids to affected areas. Patients can be given prednisone 0.5–1 mg/kg/day and up to 2 mg/kg/day if there is no improvement. After the rash resolves to grade 1 or 0, prednisone should be tapered over 4–6 weeks and ICB may be re-challenged (15). As a targeted, steroid-sparing agent, tocilizumab, an anti-IL-6R monoclonal antibody that limits Th17 differentiation and pro-inflammatory response, may be considered for persistent maculopapular rashes (32). Dupilumab, an anti-IL-4Rα monoclonal antibody that blocks signaling in Th2 pathways implicated in eczema and itch, may be considered for eczematous DAEs and for pruritus (30, 32). Omalizumab has also been shown to relieve pruritus with increased IgE (33). Per NCCN Guidelines for Management of Immunotherapy-Related Toxicities (version 1.2022), gabapentinoids, aprepitant, and narrow-band UVB phototherapy may also be considered for persistent and severe pruritus (15).

## Drug Reaction With Eosinophilia and Systemic Symptoms

While classic DRESS is rare to ICB, patients commonly present with generalized maculopapular rash, fever, and concurrent extracutaneous irAEs (transaminitis, azotemia, and colitis) mimicking classic DRESS. While rarely reported in literature (34–37), FAERS has records of 24 reported cases of DRESS with pembrolizumab, 46 with ipilimumab, 89 with nivolumab, 6 with atezolizumab, 1 with avelumab, 3 with durvalumab, and 1 with cemiplimab. In classic DRESS, grade 2 eosinophilia (≥1,500 μL^–1^) is present in up to 81% of cases and grade 1 eosinophilia (700–1,499 μL^–1^) in 14% of cases (38); however, eosinophilia is less frequently observed in irDAEs, in about 51% (32). Histopathology of the morbilliform eruption of DRESS is often non-specific and may demonstrate features such as interface dermatitis that is present in various dermatoses (16). To manage ICB-DRESS, the culprit ICB should be held initially. Due to systemic involvement, high-dose and prolonged courses of corticosteroids may be required, with a slow 6- to 8-week taper after ICB-DRESS resolution. Anti-TNF-α, tocilizumab, and dupilumab may be considered as a steroid-sparing, precision medicine approach (16, 30, 37).

## Acute Generalized Exanthematous Pustulosis

Acute generalized exanthematous pustulosis (AGEP) is an extremely rare DAE to ICB, characterized by small sterile pustules and edematous erythema. FAERS includes 11 reported cases of AGEP with pembrolizumab, 4 with ipilimumab, 6 with nivolumab, and 8 with atezolizumab. No cases of AGEP have been recorded with avelumab, durvalumab, or cemiplimab. Diagnosis is based on clinical and histopathological findings. AGEP has an acute onset, typically within 48 h of starting a new drug, and may have spontaneous rapid resolution (16, 39, 40). Biopsy reveals subcorneal pustules and subepidermal mixed cellular infiltrates with eosinophils (39, 41). Management of AGEP includes holding ICB and a combination of topical and systemic corticosteroids (oral prednisone 0.5–1 mg/kg/day (7, 16). After multi-disciplinary discussion, ICB may be resumed once AGEP has resolved to grade ≤1.

## Bullous Pemphigoid, Lichen Planus Pemphigoides, and Bullous Lichen Planus

Although rare with anti-CTLA-4 blockade, bullous disorders secondary to anti-PD-1/PD-L1 therapies have been reported with increasing frequency and may become severe. Through FAERS, 204 cases of bullous dermatitis, autoimmune blistering disease, pemphigoid, or generalized bullous fixed drug eruption have been reported with pembrolizumab. Eighty-nine cases have been reported with ipilimumab, 479 with nivolumab, 41 with atezolizumab, 5 with avelumab, 44 with durvalumab, and 16 with cemiplimab.

**FIGURE 2:**
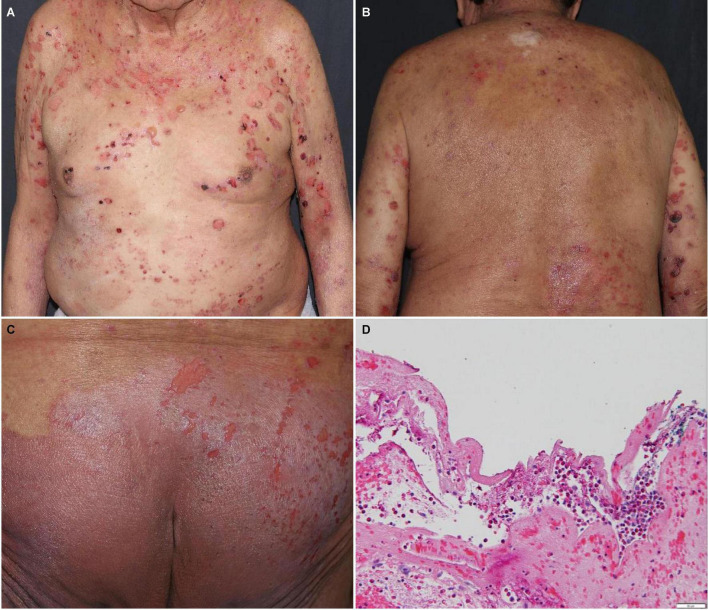
Bullous pemphigoid with tense bullae and erosions on the **(A)** trunk, **(B)** back, and **(C)** buttocks. **(D)** Subepidermal vesicular dermatitis with abundant eosinophils and fibrin. Direct immunofluorescence studies revealed linear deposits of IgG, IgG4 and C3 at the basement membrane zone of the dermal-epidermal junction, focal deposits of fibrin in the reticular dermis and deposits of fibrin in the debris within the cleft.

Bullous pemphigoid (BP) (Figure 2) is the most frequently reported bullous disorder relating to anti-PD-1/PD-L1 blockade, and often presents with prodromal or concurrent pruritus. BP commonly develops as a delayed DAE, appearing >4 months after starting anti-PD-1/PD-L1 blockade (10). BP associated with anti-PD-1/PD-L1 blockade appears to present in younger patients (median age 74 years, range: 50-93 years) and affects the mucosal membranes more frequently (in 38.1% of patients on anti-PD-1/PD-L1 blockade) than idiopathic BP (42). In one study, subepidermal blisters were seen in 81% and eosinophilic infiltrate in 82% of anti-PD-1/PD-L1 blockade associated BP cases on histopathology. Direct immunofluorescence was positive in 79% of cases for IgG deposition and 80% for C3 deposition at the basement membrane zone of the dermal-epidermal junction. BP180 and BP230 antibodies were elevated on serology in 61 and 13% of cases, respectively (42).

Compared to idiopathic BP, BP secondary to ICB may be more difficult to diagnose and manage (43). Serology with elevated BP180 antibodies and biopsy with direct immunofluorescence showing IgG and C3 deposition at the basement membrane zone of the dermal-epidermal junction are suggestive of BP (42). Unlike idiopathic BP which generally responds well to systemic steroid treatment, BP from ICB may be systemic steroid-refractory (44). CTCAE grade 1/2 BP in patients on ICB can be managed with high-dose topical steroids and low-dose systemic steroids. In more severe or refractory cases, systemic steroids can be increased to 0.5–1 mg/kg/day (28, 45). In one review, BP from ICB required discontinuation of ICB in 76% of cases (46). In lieu of continued systemic steroid use or for steroid-refractory cases, rituximab, intravenous immune globulin (IVIg), omalizumab, dapsone, dupilumab, or methotrexate can be considered (28, 47, 48).

Other high grade bullous disorders from ICB which have been less frequently observed include blistering lichenoid reactions, such as lichen planus pemphigoides and bullous lichen planus (49–51). Lichen planus pemphigoides presents with clinical features of both BP and lichen planus, with oral involvement in up to half of cases. Histopathological features can include lymphocyte-rich subepidermal bullae with margins exhibiting features of lichen planus including colloid bodies or focal vacuolar degeneration. As in BP, direct immunofluorescence can show IgG and C3 deposits along the basement membrane (49). In cases of bullous lichen planus associated with ICB, patients may present initially with lichenoid plaques that blister with onset time ranging from 3 to 8 months. Histopathology demonstrates lymphocytic infiltrate, as in lichen planus. Direct immunofluorescence may show non-linear IgM and C3 colloid bodies at the dermal-epidermal junction and BP180 antibodies are not expected to be elevated (51). Treatment of lichen planus pemphigoides in the setting of ICB can include topical steroids, systemic steroids, dupilumab, and rituximab, IVIg, as in BP (49). For CTCAE grade ≥3 lichenoid eruptions, biologics including infliximab and tocilizumab may be considered (10). For steroid-refractory bullous lichenoid DAEs, treatment with cyclosporine to inhibit T-cell activation may be used (52).

## High Grade Psoriasiform Dermatologic Adverse Events

High grade psoriasiform DAEs to anti-CTLA-4 and anti-PD-1/PD-L1 blockade have been widely reported in the literature (53–56). In FAERS, 152 cases of psoriasiform DAEs have been recorded in association with pembrolizumab, 40 with ipilimumab, 243 with nivolumab, 57 with atezolizumab, 5 with avelumab, 24 with durvalumab, and 4 with cemiplimab. In one study of 21 patients, 72% had a pre-existing history of psoriasis (53). Psoriasiform DAEs subtypes included plaque (53.3%), scalp (20.0%), guttate (20.0%) psoriasis, or sebopsoriasis (6.8%) (53). Onset from ICB initiation to psoriasis development is 90.5 ± 77.7 days for new-onset psoriasis and 32.8 ± 21.8 days for flares of pre-existing psoriasis (53). In a multicenter study of 76 patients with pre-existing psoriasis and various malignancies treated with ICB, 43 (57%) patients had a psoriasis flare after a median of 44 days after ICB initiation. Seven patients experienced grade 3–4 psoriasiform DAEs and 16 (21%) required systemic therapy. Of the 15 patients with pre-existing psoriatic arthritis prior to ICB, 6 experienced arthritis flares (56). Notably, progression-free survival was significantly longer in patients who experienced a psoriasis flare compared to those who did not (39 vs. 8.7 months, *p* = 0.049) (56). When biopsied, psoriasiform DAEs show parakeratosis, diminished granular layers, and acanthosis, with varying concomitant spongiosis (14).

Psoriasiform DAEs are thought to develop due to upregulation of Th17 lymphocytes as a result of PD-1 blockade (28). Therefore, in addition to holding ICB and using topical steroids, targeted management for psoriasiform DAEs and psoriasis flares includes anti-IL-12/23, anti-IL-23, and anti-IL-17 inhibitors, or apremilast (28, 32). Table 2 summarizes management of the aforementioned high grade DAEs associated with ICB.

**TABLE 2:** Management of high grade DAEs.

	ICB rechallenge	Recommendations	Level of evidence (66)
True SJS/TEN	Contraindicated	Stop ICB Supportive care (hydration, electrolyte management, nutrition, etc.) Dermatologic, ophthalmologic, gynecologic, and/or urologic consultations Hospital, intensive care unit, or burn unit admission for widespread desquamation and life-threatening complications Etanercept, cyclosporine, and/or IVIg	I (16, 27, 28, 37)
SJS/TEN-Like reactions	May be considered	Hold ICB Dermatologic evaluation Begin methylprednisolone/prednisone (1–2 mg/kg/day) and/or steroid-sparing therapies such as etanercept, cyclosporine, tocilizumab and/or IVIg	IV (17, 18)
Maculopapular rash	May be considered	Hold ICB High potency topical corticosteroids to affected areas on body; low potency topical corticosteroids to face/folds Prednisone 0.5–1 mg/kg/day Consider inpatient care For pruritus, consider gabapentinoids, aprepitant, dupilumab, omalizumab, or narrow-band UVB phototherapy	I (15)
DRESS	May be considered	Hold ICB Dermatologic evaluation High-dose and prolonged courses of oral or intravenous corticosteroids with slow taper Addition of steroid-sparing therapies such as anti-TNF-α, tocilizumab, dupilumab	I (16, 27, 37)
Bullous pemphigoid and lichen planus pemphigoides	May be considered	Hold ICB until grade ≤1 High potency topical corticosteroids twice daily Prednisone 0.5–1 mg/kg/day for grade ≥2 reactions For steroid-refractory or grade ≥3 reactions consider: Rituximab (375 mg/m^2^ weekly × 4 weeks) ± IVIg (1 g/kg every 4 weeks) Omalizumab (300 mg every 4 weeks) Dapsone (starting dose 25 or 50 mg daily) Dupilumab (600 mg loading dose, then 300 mg every other week) Methotrexate (15–25 mg daily with folic acid supplementation)	I (10, 28, 47, 48)
Psoriasiform DAEs	May be considered	Hold ICB until grade ≤1 Topical corticosteroids Consider targeted biologics including anti-TNF-α, anti-IL-12/23, anti-IL-23, anti-IL-17, or apremilast) Consider systemic retinoids (acitretin)	I (10, 15, 56)

## Discussion

True SJS/TEN due to ICB may be overdiagnosed (17) due to the similarity with and novelty of SJS/TEN-like reactions. Because SJS/TEN-like reactions to ICB present variably along a clinical spectrum, they have been described by various terms including: high grade lichenoid dermatosis or unclassified dermatosis (17), lichenoid mucocutaneous eruptions (57), and progressive immunotherapy-related mucocutaneous eruption (PIRME) further complicating definitive diagnosis of this pattern of reactions (18). Differentiating true SJS/TEN from DAE mimickers is integral to a patient’s cancer care, as emerging evidence suggests that although ICB challenge should not be attempted in cases of true SJS/TEN, it may be achievable after SJS/TEN-like reactions have improved (18).

Best management strategy for ICB-associated DAEs requires ongoing investigation. Evidence regarding the safety of systemic steroids for irDAE management is conflicting. Importantly, the risks and benefits of systemic corticosteroids for the management of high grade DAEs must be carefully weighed, as there is mixed evidence that systemic corticosteroid use may dampen the antitumor effects of ICB. Specifically, in patients treated with ipilimumab for melanoma, use of high-dose systemic corticosteroids was associated with significantly shorter overall survival and the time to treatment failure compared to use of low-dose corticosteroids (58). Similarly, in a study of patients treated with ICB for non-small cell lung cancer, use of systemic corticosteroids at the time of ICB initiation was significantly associated with decreased progression-free survival and overall survival (59). However, a pooled analysis of multiple phase III trials of nivolumab for advanced melanoma found no difference in objective response rates between patients who received systemic corticosteroids or other suppressive immune-modulating agents and those who did not (60).

Although steroids are currently the initial therapy for many cutaneous and extracutaneous ICB toxicities, there is increasing support for tailored approaches that account for clinical presentation and circulating biomarkers (61). In patients with DAEs associated with ICB, IL-6 has been found to be elevated in 52% of 65 patients, elafin in 30% of 43, IL-8 in 25% of 20, IgE in 24% of 101, and IFN-γ in 23% of 26 patients. Notably, serum IgE levels also correlate with DAE severity (32). In hospitalized cancer patients with high grade DAEs, elevated elafin, IL-6, and TNF-α were shown to be associated with higher all-cause mortality. As such, tocilizumab, an anti-IL-6 agent, was recently investigated and shown to be effective for management of ICB toxicities across various organ systems in 86% of 91 cancer patients without disease progression (62). Median resolution of ICB toxicity after tocilizumab initiation was 6.5 days (63). In patients with high grade DAEs associated with ICB and elevated IL-6, tocilizumab is a promising steroid-sparing agent (64). As precision medicine with targeted biologics continues to develop, future research is needed to determine its utility in the management of DAEs. In oncodermatology, continued research to explore cytokines associated with poor outcomes in cancer patients as potentially useful therapeutic targets is important (65).

Through data from the FAERS database, we show that high grade DAEs such as SJS/TEN to immunomodulatory agents are not uncommon. We expect that as these innovative anti-cancer therapies continue to be used and as new ones develop, more patients will develop high grade DAEs. Familiarization with high grade DAEs and understanding of how to manage these will result in better outcomes through prompt management of patients with life-threatening cutaneous adverse reactions such as SJS/TEN, DRESS, and AGEP, and ability to rechallenge and continue ICB in patients with mimickers of SJS/TEN such as SJS/TEN-like reactions, bullous pemphigoid, lichenoid planus pemphigoides, and bullous lichen planus. Further research must be done not only to better delineate the high grade DAEs associated with ICB use but also to identify effective management strategies via precision medicine that do not reduce ICB efficacy.

## Author Contributions

AK and AM contributed to all parts of the conception, design, and writing of the manuscript. Both authors contributed to the article and approved the submitted version.

## Conflict of Interest

AM received research funding from Incyte Corporation and Amryt Pharma, and royalties from UpToDate and sits on the advisory board for Alira Health, Blueprint Medicines, Janssen, and Protagonist therapeutics. The remaining authors declare that the research was conducted in the absence of any commercial or financial relationships that could be construed as a potential conflict of interest.

## Publisher’s Note

All claims expressed in this article are solely those of the authors and do not necessarily represent those of their affiliated organizations, or those of the publisher, the editors and the reviewers. Any product that may be evaluated in this article, or claim that may be made by its manufacturer, is not guaranteed or endorsed by the publisher.
